# Local Treatment of Lung Cavities

**Published:** 1874-01

**Authors:** Fr. Mosler

**Affiliations:** Griefswald


LOCAL TREATMENT OF LUNG CAVITIES.—*By Prof. Fr. Mosler (Griefswald.)*—It has long been the endeavor on the part of a number of physicians to effect a local treatment of lung diseases, especially of lung cavities.

Since the time when we have learned that every lung inflamation, may, under certain conditions, lead to caseous degeneration and suppuration of the lung parenchyma, since the infectious material has been demonstrated, we find ourselves driven to attempt the local treatment of all these affections. Wherever the secretion of the diseased part reaches, new deposits arise, whether in the lung sor other organs. Quite recently I had a case of caseous pneumonia in my clinic and the patient could not be induced to expectorate his sputa, but continued to swallow it constantly; in a short time he died of consequent intestinal affection.

I have used with advantage in different bronchial and lung affections, besides expectorants, the much recommended inhalations of carbolic acid. But, unfortunately, many cases are not benefited to the measure of our expectation. I made the attempt, then, last year *to introduce remedies into the lung cavities from the outside, through the walls of the throax.*

My first attempt was in a case of phthisis in the last stage of the disease. I only wished to demonstrate the possibility of the method. It was the case of a laborer, aged fifty-one, affected with right-sided pneumonia since 1869. He had had several attacks of hemoptysis and had become emaciated in extreme degree. In the right apex and in front was a superficial cavity reaching down to the fourth rib and of easy demonstration.

On Nov. 1, 1872, I pushed through the thorax wall, second intercostal space, six ctm. from the right border of the sternum, a tolerably large canula of the well known aspiration syringe o

Thiersch. I then injected through this canula 20 ccm. of a dilute solution of the permanganate of potash. The syringe was then unscrewed from the canula, which was permitted to remain, and on the three following days the injection, same quantity, was repeated. On the fourth day the canula became occluded and was removed. *The patient suffered, meanwhile, not the least inconvenience.* I considered myself justified in repeating this experiment in another way, *in order to give the secretion free exit.*

In the case of a painter, aged forty-nine, who had been treated in my clinic for five years for a bronchiectic cavity of the right upper lobe, I made an opening into the very superficial cavity; in the following way :

A long incision (3 ctm.) dividing the skin and superficial intercostal muscles, commencing about 5½ ctm. from the right border of the sternum, was made along the upper border of the third rib; the wall of the cavity was gradually opened with a suitable pair of forceps. A whistling sound in inspiration, the escape of a purulent secretion mingled with air bubbles furnished the certain proof that the cavity had been opened. A pretty large silver drainage tube was introduced into the cavity and fastened with plaster to the wall of the chest. Pledgets of carbolized lint and an ice bladder were then applied. *Pus flowed through the canula abundantly, particularly on coughing.*

On the tenth day a hemoptysis, perhaps as the result of granulation formation in the cavity. A dilute solution of liquor ferri sesquichloridi was blown through the canula, whereupon the hemorrhage soon ceased. Subsequently, a dilute solution of carbolic acid and tincture of iodine was brought into the cavity, twice daily, by the same *pulverisateur.* He declared that he felt it enter the cavity.

*The pus had now assumed a laudable character and was less profuse.* There could be no doubt that this disinfection had a better result than the inhalation of carbolic acid by the mouth.

I believe I am justified in one statement, however, as a certain conclusion from these experiments, viz., *that the local treatment of lung cavities can be effected.*

As to the final conclusion concerning the value of this operation, opinions will differ. There can be no doubt that my experiments have proven one fact, viz: That the lung is more tolerant of external manipulation; that these attacks are less dangerous and more easily executed than has been heretofore believed.

*Addendum.*—Three months afterwards, October 1st, the condition of the last mentioned patient had so far changed for the worse that he continued to lose strength in spite of nourishment and stimulation. Pus continued to flow freely through the canula, on which account I continued the insufflation through the canula of dilute carbolic acid twice daily. He died with manifestations of cardiac paralysis, on October 5. The *post-mortem* in this case cannot fail to be of extreme interest.

After opening the thorax, the left lung retracted well; the layers of the right pleuræ were perfectly adherent. The right lung in its whole extent, most markedly from the third rib upwards, was solidly adherent to the pleura costalis. At the lower lobe is a canal running obliquely from before and below, backwards and upwards with smooth walls; this canal leads to a cavity which takes up the greater part of the upper lobe. The cavity is filled with yellowish purulent fluid. The inner wall of the cavity is marked by projecting prominences differing in their reddish color from other parts of the wall of the cavity, which have a grayish black color and smooth surface. The prominences show distinctly in places a feebly granulated surface.

